# Artificial Neural Network-Based Failure Pressure Prediction of API 5L X80 Pipeline with Circumferentially Aligned Interacting Corrosion Defects Subjected to Combined Loadings

**DOI:** 10.3390/ma15062259

**Published:** 2022-03-18

**Authors:** Suria Devi Vijaya Kumar, Saravanan Karuppanan, Mark Ovinis

**Affiliations:** Mechanical Engineering Department, Universiti Teknologi PETRONAS, Bandar Seri Iskandar 32610, Perak Darul Ridzuan, Malaysia; saravanan_karuppanan@utp.edu.my (S.K.); mark_ovinis@utp.edu.my (M.O.)

**Keywords:** artificial neural network, finite element analysis, corrosion assessment method

## Abstract

Conventional pipeline corrosion assessment methods produce conservative failure pressure predictions for pipes under the influence of both internal pressure and longitudinal compressive stress. Numerical approaches, on the other hand, are computationally expensive. This work provides an assessment method (empirical) for the failure pressure prediction of a high toughness corroded pipe subjected to combined loading, which is currently unavailable in the industry. Additionally, a correlation between the corrosion defect geometry, as well as longitudinal compressive stress and the failure pressure of a pipe based on the developed method, is established. An artificial neural network (ANN) trained with failure pressure from FEA of an API 5L X80 pipe for varied defect spacings, depths, defect lengths, and longitudinal compressive loads were used to develop the equation. With a coefficient of determination (R^2^) of 0.99, the proposed model was proven to be capable of producing accurate predictions when tested against arbitrary finite element models. The effects of defect spacing, length, and depth, and longitudinal compressive stress on the failure pressure of a corroded pipe with circumferentially interacting defects, were then investigated using the suggested model in a parametric analysis.

## 1. Introduction

Pipelines are the preferred transport medium for hydrocarbons since they are cost-effective and secure. Regardless of the maintenance and preventative strategies used, pipelines will inevitably deteriorate over time, compromising its integrity. Hence, the integrity of a pipeline must be assessed regularly, to ensure that it can sustain operational pressures. Otherwise, catastrophic failures may occur causing operations to be disrupted [1]. Various corrosion assessment methods, such as the DNV-RP-F101 and ASME B31G, are available to assess the integrity of pipelines periodically.

Single corrosion defects and interacting corrosion defects are the two most prevalent forms of corrosion categories. A single corrosion defect is one that is isolated and does not interact with surrounding defects [1]. Interacting corrosion defects, on the other hand, are described as corrosion defects that interact with surrounding defects. This research focuses on interacting corrosion defects that are circumferentially aligned. Circumferentially aligned interacting corrosion defects are those that are separated by full-wall thickness in the pipe’s hoop direction.

Circumferential interacting defects are more complex compared to single defects due to the interaction amongst them. The failure pressure is significantly less than that caused by a single defect. The presence of a defect causes a disturbance in the hoop stress distribution in a pipeline. The region that is affected by this disturbance in stress and strain fields of the pipeline is called area of influence of defect. When adjacent defects are present, the area of influence of the defects overlap. As such, the failure pressure of the pipe with interacting corrosion defect is lower compared to the failure pressure of single defects [2,3,4].

### 1.1. Overview of Corrosion Defects

**Figure 1:**
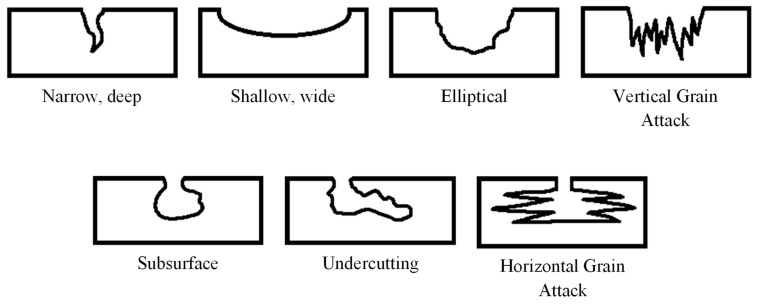
Types of pitting corrosion [9].

Corrosion is the deterioration of a metal because of electrochemical interactions between a material and its environment. Localized corrosion is one of the most harmful types of corrosion in hydrocarbon pipelines because the metal deteriorates at a rapid rate [5]. This type of corrosion causes the material to crack or perforate, resulting in rapid structure collapse [6]. There are several forms of localized corrosion in pipelines. Pitting corrosion, as illustrated in Figure 1, is one of the most destructive forms of corrosion [7,8].

**Figure 2:**
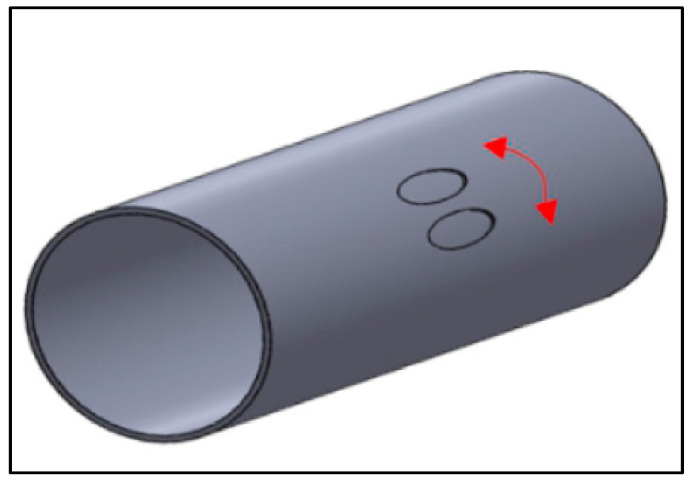
Circumferential interacting defects [15].

In the presence of two corrosion defects in close proximity, an interaction between the defects exist and this interaction is influenced by the spacing between the defects [10,11,12,13]. Circumferentially aligned corrosion defects are separated by full wall thickness in the circumferential direction of the pipe, as illustrated in Figure 2. As the defect spacing is increased, the overlap region of influence decreases causing the failure pressure of the pipeline to increase. Beyond a certain defect spacing the interaction between the two defects ceases to exist as the overlap region is no longer present [10,13,14]. This point is known as the interaction limit. Beyond the interaction limit, the defects are treated as isolated defects (single defects).

This condition is more severe in the presence of compressive stress in the longitudinal direction, which causes the build-up of strain at the location of defect (critical zone) [16]. Premature failure of the pipe occurs when the stress at the critical point exceeds the material’s true ultimate tensile strength. As such, to maintain a safe and efficient operation, the integrity of a pipeline must be evaluated from time to time [17,18,19].

### 1.2. Conventional Pipeline Integrity Assessment Methods for Corroded Pipes

It has been well established that interacting defects cause more harm to a pipeline than a single defect, as it significantly reduces the failure pressure of the pipeline [1,11,13,20,21,22,23,24]. In most cases, pipelines are subjected to both internal pressure and longitudinal compressive stress, due to the temperature of the transported fluid, Poisson’s effect, as well as ground topology and movement.

Various corrosion assessment methods have been developed to assess the integrity of a pipeline under various conditions to ensure safe operations. Failure pressure predictions based on conventional corrosion assessment codes such as the ASME B31G, Modified B31G, SHELL 92, RSTRENG, PCORRC, and DNV-RP-F101, are conservative, leading to premature pipeline repairs and replacements [25]. Of all the codes that are being used in the industry for failure pressure prediction of corroded pipes subjected to combined loading, the DNV-RP-F101 code (DNV) is most comprehensive [23,26,27,28,29,30].

Over the years, various studies have been carried out to develop accurate interaction rules based on corrosion defect categories such as longitudinal interacting defects, circumferential interacting defects, or single defects. The interaction rule applied for circumferentially aligned interacting defects in commonly used corrosion assessment codes in the industry are presented in Table 1.

Among the assessment methods used in the industry, DNV is known to be the most comprehensive method. However, since the validation of the DNV approach was based on burst tests carried out on pipes of grades API 5L X45 to API 5L X65, it was designed primarily to examine the integrity of medium toughness pipelines. When applied to a high toughness pipelines, it results in inaccurate and overly conservative predictions [3,27]. Besides, this method is only applicable for interacting defects (longitudinal and circumferential) subjected to internal pressure only.

### 1.3. Finite Element Method as a Reliable Pipeline Failure Pressure Prediction Tool

As current corrosion assessment methods for high toughness pipelines are conservative, the finite element method (FEM) is widely applied. FEM allows non-linear structural analysis, as structures that are subjected to large displacements result in geometrical change. This enables accurate prediction of failure pressure, as it accounts for strain hardening, and plastic and elastic deformation of the material [32,33].

The failure pressure accuracy evaluated using FEM is heavily dependent on the right selection of model features, boundary conditions, and material parameters [14]. The model’s meshing size has a significant impact on the result accuracy. Meshes that are smaller in size produce higher accuracy but consume greater time to simulate. As such, optimization of the pipe model is important to reduce simulation time while maintaining the result accuracy [34]. If any of the components are inadequately specified, the FEM findings will be incorrect, resulting in incorrect failure pressure estimates. As such, before beginning a large-scale parametric investigation utilizing FEM, the method must first be validated against full-scale burst tests. Various studies have proven that FEM results in predictions that are close to real-world data [3,13,26,27,32]. In addition, it has been categorized as a Level 3 evaluation method for pipeline failure pressure prediction [24].

Quarter models with symmetric boundary conditions (to treat the pipe as a whole) are commonly used to shorten the computation time of a finite element (FE) simulation. Nevertheless, a single simulation run could take several hours. As a result, utilising FEM for parametric studies to estimate the corroded pipe failure pressure subjected to combined loading is time consuming. In time-critical scenarios, a quicker response time means prevention of disastrous failures, reduced damage and cost of repair.

FEM has been widely applied for predicting the failure pressure or residual strength of a corroded pipeline. Lee et al. [citation] utilised FEM to assess the failure of an API 5L X65 gas pipe at multiple corrosion defect regions. Belachew et al. [32] utilised FEM for burst test analysis of a corroded API 5L X52 grade steel pipe. It was found that commercial codes for pipeline capacity assessment are conservative. Similarly, Arumugam et al. [26] utilised FEM for failure pressure analysis and development of equations to predict the failure pressure of API 5L X52 pipelines with single corrosion defects.

The training data in the study conducted by Xu et al. [35] was generated using FEM for the development of an ANN to predict the failure pressure of a corroded pipe. FEM could predict failure pressure of pipelines with a relative error percentage of less than 1%. As such, it can be said that FEM results in accurate predictions, and hence, a reliable method to obtain training data for the development of an ANN.

### 1.4. Application of Artificial Neural Network as Corroded Pipeline Failure Pressure Prediction Tool

An ANN is an interconnected collection of simple processing elements called nodes, with processing capability of the network stored in the inter-unit connection strengths called weights, trained by learning a set of data patterns [36]. An ANN can learn, recognise, and make inferences from complex, non-linear data without instructions [37]. It can learn from a set of training data (including noisy data). Weights and biases are imposed to neurons in the neural network layers. They are reinitialized until the network gives accurate results [38]. It is very important that the training dataset has enough inputs and outputs to make the neural network’s output accurate. [39].

For failure pressure assessment, the use of ANN has improved with time, resulting in improved practical applications. Researchers considered the physical, operational, and mechanical elements that impact a pipeline’s residual strength in early ANN implementations [40]. Obtaining large training datasets for such scenarios, however, is difficult. In their study, Shirzad et al. [41] and Senouci et al. [42] emphasised that without reliable data, an ANN with adequate accuracy and robustness cannot be obtained.

In this approach, the issue of having a limited amount of reliable data (usually burst tests) can be overcome using FEM to generate training data for the ANN model [3,33]. In a study conducted by Xu et al. [12], a feed forward neural network was utilised with a backpropagation algorithm to predict the corroded (interacting defects) pipe failure pressure. FEM was used to generate training data for the neural network. Four neurons were used in the input layer, five neurons in the hidden layer, and one neuron in the output layer. The input parameters are the normalized defect length, depth, longitudinal spacing, and circumferential spacing. The number of neurons in the hidden layer was calculated using Equation (1), where $N_{h}$ is the number of hidden neurons, $N_{i}$ is the number of inputs while $N_{o}$ is the number of outputs. The neural network was validated against burst tests, and it was found that the ANN can predict the failure pressure of a corroded pipe to a high accuracy.

(1) $$N_{h}=2\sqrt{N_{i}+N_{o}}$$

In this study, an empirical equation for the failure pressure prediction of a corroded API 5L X80 pipeline with circumferentially aligned interacting corrosion defects subjected to internal pressure and longitudinal compressive stress was developed. Then, a correlation between the geometry of a defect and the pressure at which it failed in an API 5L X80 pipeline was determined. As for the empirical equation, weights and biases of an ANN trained with FEM data were used to develop the equation. The equation was then tested against arbitrary FE models.

## 2. Methodology

### 2.1. Geometric Parameters Overview

To generate data for the training of the ANN, FEA was conducted based on the parameters shown in Table 2. The manipulated parameters are the length and depth of the defect, spacing between defects, and longitudinal compressive stress acting on the pipe. These parameters are presented as normalised values. The corrosion defect width was not considered, as its influence on the failure pressure of a corroded pipeline subjected to combined loadings is minimal [1,24,26].

### 2.2. Development and Validation of Finite Element Method

#### 2.2.1. Development of Finite Element Method

**Figure 3:**
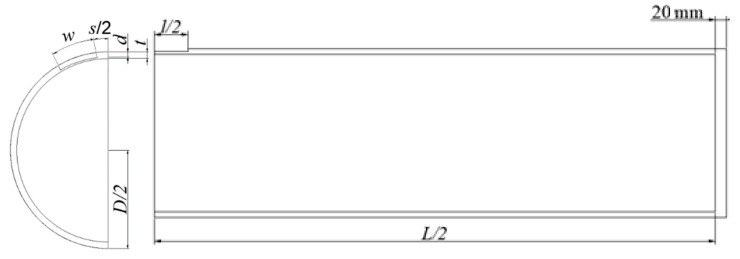
A quarter pipe model of a pipe with circumferential interacting corrosion defects.

The process of FEM development was divided into four steps, modelling of the pipe with corrosion defects, meshing of the pipe model, application of boundary conditions, and determination of the failure criterion. During the modelling process, a few design features were identified and set to ensure an efficient FE simulation. The model features and justification are listed in Table 3. The schematic diagram of the quarter model is illustrated in Figure 3.

The quarter models were meshed with solid elements due to the thickness of the pipe wall. The pipe body and defect region were meshed using hexahedral SOLID185 components, while the rigid body was meshed using tetrahedral SOLID186 elements. SOLID185 is characterised by eight nodes with three degrees of freedom, whereas SOLID186 can accommodate curved boundaries (irregular shapes). Both elements accommodate creeping, high stress stiffening, plasticity, significant deflection, swelling, and high strain [46].

According to the British Standards Institution (BSI) recommendations, the defect zone number of layers was set to three with a length and depth of 2 mm [47]. A convergence test was performed prior to finalising these mesh settings to achieve the least possible computation cost loss of accuracy. The convergence test results are summarised in Table 4. Based on the results, the optimal number of mesh layers in the defect region was determined to be three.

A 0.5 mesh bias (aspect ratio) was added to the elements moving away from the defect location, with 80 divisions. As the region away from the defect does not require a fine mesh, the mesh bias was used to save computation time without jeopardizing the results accuracy.

**Figure 4:**
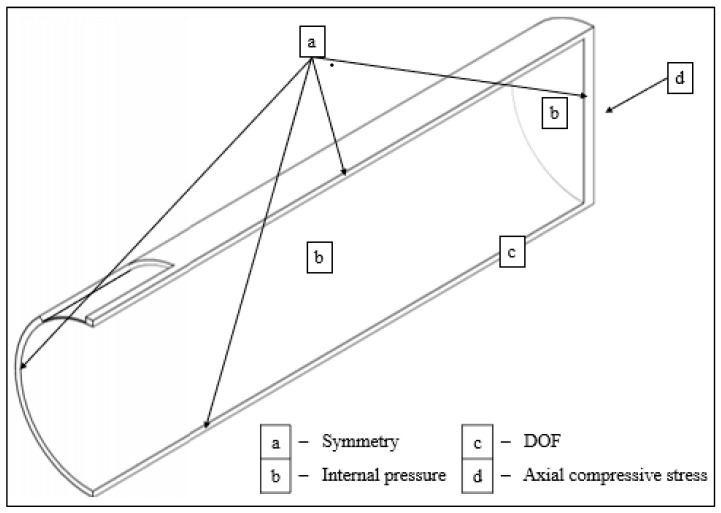
Application of symmetrical boundary conditions, internal pressure, compressive stress, and degrees of freedom (DOF) constraint for quarter pipe models.

Figure 4 shows symmetric boundary conditions for each quarter model. *X*, *Y*, and *Z* axis constraints (Figure 4) were imposed to the quarter model length away from the area of interest to prevent unwanted rigid body movements. The inner surface of the pipe and the outer surface of the rigid body (Figure 4) were subjected to incremental ramped loading and transient analysis. Timesteps regulated these loads. For pipes only subjected to internal pressure, the pressure was applied incrementally during the first timestep. For combined loadings, a longitudinal compressive stress was applied during the first timestep, and internal pressure was applied during the second timestep.

The von Mises stress-based failure criterion used in this study considers the local stress in the defect region. The material fails when the von Mises stress exceeds the true ultimate tensile strength, ${\sigma *}_{UTS}$ [45,48,49]. The effective stress, $\sigma_{e}$, is a function of hoop, $\sigma_{h}$, radial, $\sigma_{r}$, and longitudinal stress, $\sigma_{l}$, as represented in Equation (2) [26].

(2) $$\sigma_{e}=\sqrt{\frac{1}{2}\left[{\left(\sigma_{h}-\sigma_{r}\right)}^{2}+{\left(\sigma_{h}-\sigma_{l}\right)}^{2}+{\left(\sigma_{r}-\sigma_{l}\right)}^{2}\right]}$$

#### 2.2.2. Validation of Finite Element Method

Prior to conducting FEA, the accuracy of the method was established by validating it against burst tests. Burst test data from Bjorney et al. and Benjamin et al. were used for the validation [50,51]. The details of the burst tests for a single corrosion defect subjected to internal pressure only conducted by Bjorney et al. is summarised in Table 5 and Table 6. As for interacting defects subjected to internal pressure, the details of the burst tests conducted by Benjamin et al. is summarised in Table 7 and Table 8.

Based on the validation results, the highest error percentage observed was 5.92% for single defects and 2.46% for interacting defects. Positive error percentage corresponds to overestimated values while underestimated values are indicated by negative values. This approach results in minimal conservatism of approximately 6%. As such, FEM is a dependable tool for predicting failure pressure to be used as input for ANN training.

The intact pressure using FEM and maximum hoop stress theory (Equation (3)) also indicated a strong correlation among the methods. The theoretical intact pressure of a pipe is 51.3 MPa, while the intact pressure determined by FEM was 50.32 MPa, amounting to a percentage error of −1.9%.

(3) $$P_{i}=\frac{\sigma_{UTS}\;t}{r_{i}}$$

### 2.3. Development and Validation of Artificial Neural Network (ANN)

#### 2.3.1. Development of Artificial Neural Network

The process of ANN development was divided into three steps, architecture determination, training of the model, and validation of the model. A feed-forward neural network was modelled to map the relationship between the input and output of a set of training data for the failure pressure prediction of a pipe with circumferentially aligned interacting corrosion defects using MathWorks MATLAB R2021b. The architecture of the network consisted of five inputs and one output which is the normalized failure pressure of the pipe. A convergence test based on the coefficient of determination (R^2^) of the ANN was carried out to optimize the number of hidden layers and neurons in each hidden layer of the ANN.

The training of the ANN utilized the Levenberg–Marquardt back-propagation algorithm. The algorithm requires less computational time to optimize the weights and biases of an ANN model, without compromising on the accuracy of the results [52]. The activation functions of neurons in the hidden layers and output neuron are represented by a hyperbolic tangent function (Equation (4)) and a linear function (Equation (5)), respectively. The training data for the ANN was obtained from the FEA.

(4) $$a\left(x\right)=\frac{2}{\left(1+e^{-2x}\right)-1}$$

(5) f(x)=x

#### 2.3.2. Validation of the Artificial Neural Network

The accuracy of the ANN is assessed by the model’s coefficient of determination (R^2^). The R^2^ value is between 0.0 and 1.0, with a higher value suggesting a better fit, which is the distance between the fitted line in the ANN’s regression plot and the training data points.

### 2.4. Material Properties

**Figure 5:**
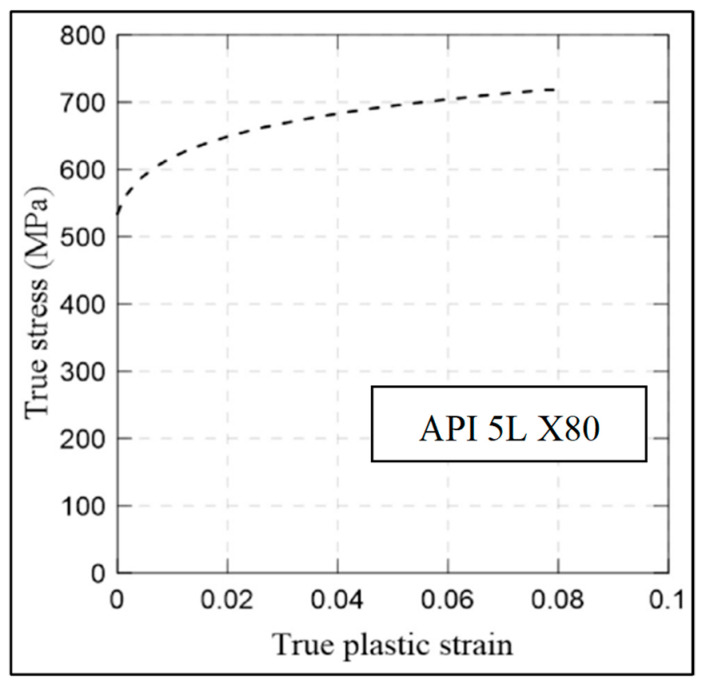
True stress-strain curve for API 5L X80.

The non-linear structural analysis used rate-independent plasticity models based on the von Mises yield criterion and isotropic hardening rule. The study used an API 5L X80 high-toughness pipe grade, and the mechanical parameters of the materials are listed in Table 9. The modulus of elasticity of the rigid end plate was set at 200 TPa with a Poisson’s ratio of 0.3. The endplate was assumed to be a hard body and the material property of the pipe body are represented by a non-linear true stress-strain curve, as depicted in Figure 5. To account for a high degree of material non-linearity of the pipe body, stress stiffening, large strains, and displacements were considered.

## 3. Results and Discussion

### 3.1. Generation of ANN Training Data Using Finite Element Analysis (FEA)

In this study, FEA was conducted to predict the failure pressure of API 5L X80 pipes. The failure pressures obtained using FEM were normalised by the intact pressures of the pipe, which was obtained using FEM as well. The failure pressures obtained are tabulated in Table 10, where the normalised defect depth, length, longitudinal compressive stress, are represented by d/t, l/D, and $\sigma_{c}/\sigma_{y}$, respectively.

### 3.2. Development of the ANN Model

**Figure 6:**
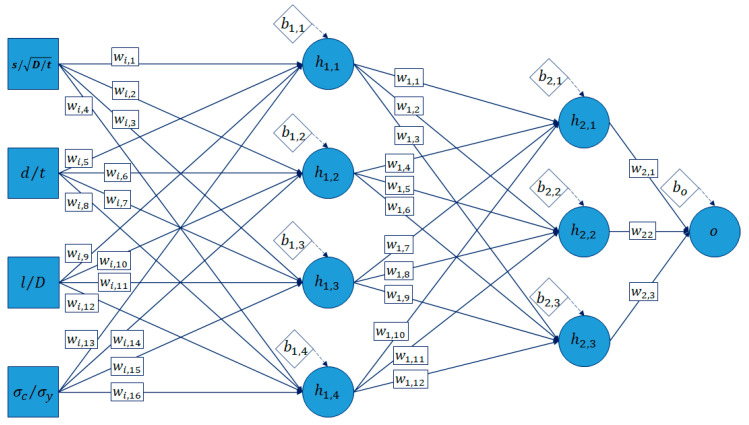
Architecture of the model CID7.

The ANN model developed using MathWorks MATLAB R2021b was a feed forward neural network using back-propagation algorithm. Based on the convergence test (Table 11) to optimise the number of hidden layers and neurons in each hidden layer, model CID7 resulted in the highest R^2^ value with a minimum number of neurons. This was to ensure that the complexity of the developed equation was as minimal as possible, without compromising on the accuracy of the results. Model CID7 consisted of two hidden layers with four and three neurons in the first and second hidden layers, respectively, as illustrated in Figure 6.

**Figure 7:**
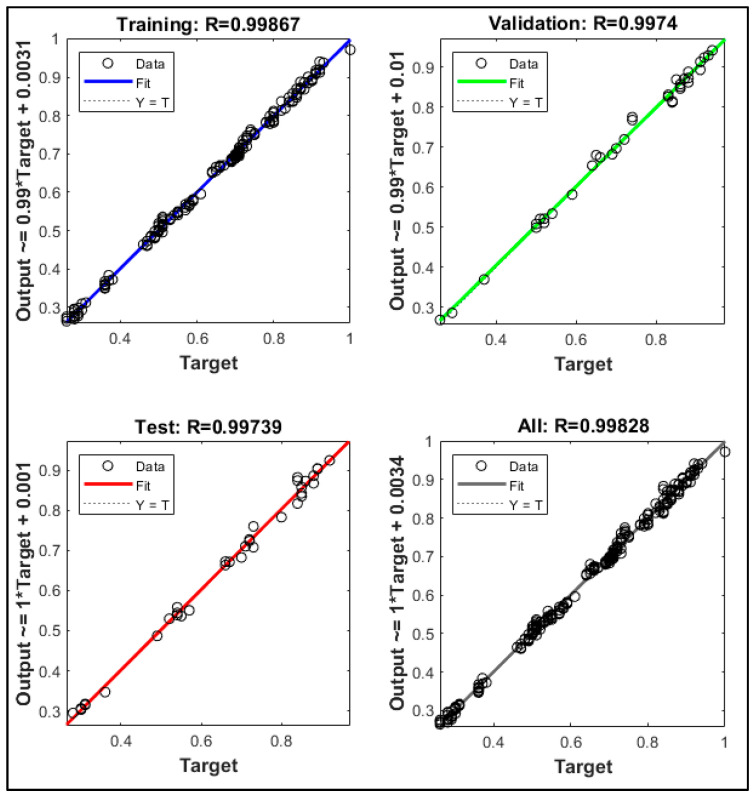
Regression plot extracted from MathWorks MATLAB R2021b.

The ANN was developed to produce an output based on four input variables. The input variables are normalised defect spacing, depth and length, and normalised longitudinal compressive stress. The output of the ANN model is the normalised failure pressure of the pipe. The performance of the ANN model was evaluated using a regression plot, as shown in Figure 7. Based on Figure 7, the capability of the model to produce results similar to that of the training data is indicated by the overlapping solid and dashed lines in the plots.

### 3.3. Development of the Empirical Equation

Model CID7 can be represented in matrix form as shown by Equations (6)–(8). This matrix was used in the development of the empirical solution for the failure pressure prediction of a corroded pipe with circumferential corrosion defects.

(6) $$\left[\begin{array}{c} h_{1,1}\\ h_{1,2}\\ h_{1,3}\\ h_{1,4} \end{array}\right]=\left[\begin{array}{cccc} 0.0494 & -0.1316 & 0.2312 & 0.0051\\ -0.2743 & 0.6362 & -0.9373 & -0.0103\\ 0.1498 & -0.3994 & 0.5559 & 0.0069\\ 0.1237 & -1.3905 & -1.9125 & 3.0428 \end{array}\right]\left[\begin{array}{c} {\left(s/\sqrt{D/t}\right)}_{n}\\ {\left(d/t\right)}_{e_{n}}\\ {\left(l/D\right)}_{e_{n}}\\ {\left(\sigma c/\sigma y\right)}_{n} \end{array}\right]+\left[\begin{array}{c} -0.0300\\ 1.0942\\ -1.1065\\ -2.9796 \end{array}\right]$$

(7) $$\left[\begin{array}{c} h_{2,1}\\ h_{2,2}\\ h_{2,3} \end{array}\right]=\left[\begin{array}{cccc} 73.6106 & -71.7986 & -202.7229 & -0.4295\\ -1.7927 & 3.0996 & 7.6167 & -0.0105\\ -4.0536 & 14.6883 & 33.5759 & -0.0707 \end{array}\right]\left[\begin{array}{c} a\left(h_{1,1}\right)\\ a\left(h_{1,2}\right)\\ a\left(h_{1,3}\right)\\ a\left(h_{1,4}\right) \end{array}\right]+\left[\begin{array}{c} -100.9641\\ 2.6968\\ 15.1230 \end{array}\right]$$

(8) $$o_{n}=\left[\begin{array}{ccc} 0.2287 & 34.9960 & -2.4594 \end{array}\right]\left[\begin{array}{c} a\left(h_{2,1}\right)\\ a{(h}_{2,2})\\ a{(h}_{2,3}) \end{array}\right]+\left[24.7446\right]$$

The input values of the ANN are normalised by the ANN model, so that the other inputs do not dominate the response. Hence, the inputs need to be normalised using Equation (9) before being used. Likewise, the output values that were used to train the ANN model were normalised to range between the values −1 and 1. Hence, the final output, o, has to be denormalized using Equation (10) to obtain the failure pressure of the corroded pipe.

(9) $$i_{n}=\frac{\left(i_{n,\;max}-i_{n,\;min}\right)\left(i-i_{min}\right)}{\left(i_{max}-i_{min}\right)}+i_{n,\;min}$$

(10) $$o=\frac{\left(i_{n}-i_{n,\;min}\right)\left(i_{max}-i_{min}\right)}{\left(i_{n,\;max}-i_{n,\;min}\right)}+i_{min}$$

The equations used to predict the failure pressure of corroded pipeline with circumferentially aligned corrosion defects are summarised in the following steps:

Step 1: Calculation of the normalised effective length and depth of defect

(11) $${\left(l/D\right)}_{e}=\frac{l_{1}+\left(s_{1}+l_{2}\right)}{D}$$

(12) $${\left(d/t\right)}_{e}=\frac{\;\left(\frac{d_{1}l_{1}+d_{2}l_{2}}{l_{1,2}}\right)}{t}$$

Step 2: Normalisation of input parameters

(13) $${\left(s/\sqrt{D/t}\right)}_{n}=\frac{2{\left(s/\sqrt{D/t}\right)}_{i}}{2}-1$$

(14) $${\left(l/D\right)}_{e_{n}}=\frac{2{\left(l/D\right)}_{e}}{1.97}-1$$

(15) $${\left(d/t\right)}_{e_{n}}=2.5{\left(d/t\right)}_{e}-1$$

(16) $${\left(\sigma c/\sigma y\right)}_{n}=\frac{2{\left(\sigma c/\sigma y\right)}_{i}}{0.6}-1$$

Step 3: Calculation of the normalised output value

(17) $$\left[\begin{array}{c} h_{1,1}\\ h_{1,2}\\ h_{1,3}\\ h_{1,4} \end{array}\right]=\left[\begin{array}{c} 0.0494{\left(s/\sqrt{D/t}\right)}_{n}-0.1316{\left(l/D\right)}_{e_{n}}+0.2312{\left(d/t\right)}_{e_{n}}+0.0051{\left(\sigma c/\sigma y\right)}_{n}-0.0300\\ -0.2743{\left(s/\sqrt{D/t}\right)}_{n}+0.6362{\left(l/D\right)}_{e_{n}}-0.9373{\left(d/t\right)}_{e_{n}}-0.0103{\left(\sigma c/\sigma y\right)}_{n}+1.0942\\ 0.1498{\left(s/\sqrt{D/t}\right)}_{n}-0.3994{\left(l/D\right)}_{e_{n}}+0.5559{\left(d/t\right)}_{e_{n}}+0.0069{\left(\sigma c/\sigma y\right)}_{n}-1.1065\\ 0.1237{\left(s/\sqrt{D/t}\right)}_{n}-1.3905{\left(l/D\right)}_{e_{n}}-1.9125{\left(d/t\right)}_{e_{n}}+3.0428{\left(\sigma c/\sigma y\right)}_{n}-2.9796 \end{array}\right]$$

(18) $$\left[\begin{array}{c} h_{2,1}\\ h_{2,2}\\ h_{2,3} \end{array}\right]=\left[\begin{array}{c} 73.6106a\left(h_{1,1}\right)-71.7986a\left(h_{1,2}\right)-202.7229a\left(h_{1,3}\right)-0.4295a\left(h_{1,4}\right)-100.9641\\ -1.7927a\left(h_{1,1}\right)+3.0996a\left(h_{1,2}\right)+7.6167a\left(h_{1,3}\right)-0.0105a\left(h_{1,4}\right)+2.6968\\ -4.0536a\left(h_{1,1}\right)+14.6883a\left(h_{1,2}\right)+33.5759a\left(h_{1,3}\right)-0.0707a\left(h_{1,4}\right)+15.1230 \end{array}\right]$$

(19) $$a\left(o_{n}\right)={0.2287a(h}_{2,1})+{34.9960a(h}_{2,2})\;-\;{2.4594a(h}_{2,3})+24.7446$$

The values of $a\left(h_{1,1}\right)$ to $a\left(h_{2,3}\right)$ are calculated using Equation (21).

(20) $$a\left(h_{x,y}\right)=\frac{2}{1+e^{-2\left(h_{x,y}\right)}}-1$$

Step 4: Denormalization of output value, $P_{nf,Eq}$

(21) $$P_{nf,Eq}=0.38o_{n}+0.62$$

Step 5: Calculation of failure pressure, $P_{f,Eq}$

(22) $$P_{i}=\frac{{\sigma *}_{UTS}\;t}{r_{i}}$$

(23) $$P_{f,Eq}=(P_{nf,Eq}*P_{i})$$

### 3.4. Evaluation of the Developed Empirical Equations

As the newly developed equations were extracted from the neural network, the new method’s R^2^ value is identical to that of the ANN, which is 0.9999. This suggests that the method produces predictions of failure pressures that are highly similar to those produced using FEA. Table 12 compares the intact pressures obtained using the three approaches. There is a strong correlation between the three approaches, with a percentage error of −0.02% between the failure pressure obtained using the maximum hoop stress and the empirical equation, and 0.68% between the failure pressure acquired using FEM and the empirical equation.

**Figure 8:**
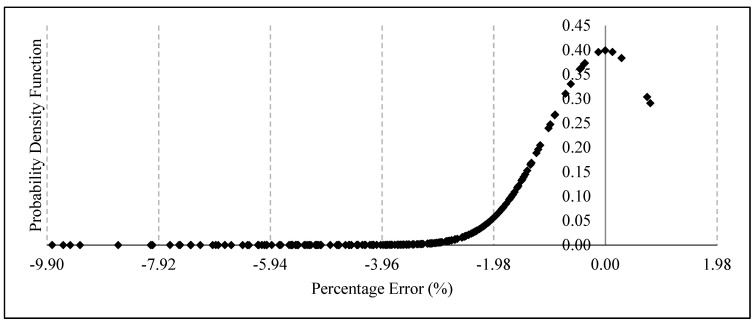
Probability distribution of the error obtained using the developed empirical equations based on FEA.

The error percentage between the estimated failure pressure using FEM and the empirical equation for the parameters used to train the ANN is shown in Figure 8. The error percentage are within the range of −9.81% to 0.80%, with a standard deviation of 1.98. The error percentages are within five standard deviations of the mean, indicating that this method is capable of predicting the failure pressure of a corroded pipe with geometric parameters within the range used to train the ANN model. The probability of obtaining a failure pressure with a percentage error larger than −9.90 % is 1 in 1,744,278. Only 1.66 % of the 241 datasets were overestimated. The overestimation, however, is negligible as it is less than 0.80%. Hence, it can be said that this method is reliable.

In the absence of burst tests of API 5L X80 pipes subjected to combined loadings for interacting defects, as a case study, FEM was used for the failure pressure prediction of API 5L X80 to further validate the equation. Table 13 summarises the parametric data, the failure pressure predictions using FEM and the empirical equation, and the error percentage between the approaches.

The error percentage between these two methods, as shown in Table 13, is between −7.60% and 0.19%. The predicted failure pressures are within the six-sigma range of 9.90%. This is valid only for normalised defect spacings within 0.00 and 2.00, normalised defect lengths within 0.00 and 0.80, normalised defect depths within 0.00 and 0.80, and normalised longitudinal compressive stress values within 0.00 and 0.60.

### 3.5. Extensive Parametric Studies

An extensive parametric study was conducted on an API 5L X80 pipe with circumferentially interacting corrosion defects utilising the empirical equation to understand the influence of defect spacing, defect depth, defect length, and longitudinal compressive stress on the pipe failure pressure. The graphs below depict the results of the study. In this parametric analysis, the failure pressures of pipes subjected to internal pressure only were utilised as reference data.

**Figure 9:**
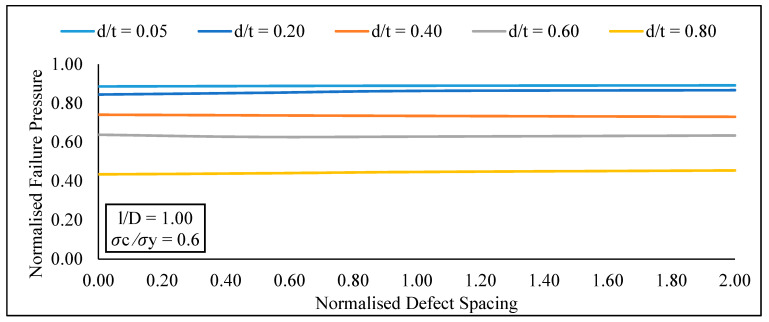
Normalized failure pressure against normalized defect spacings for various normalised defect depths at constant normalized defect length and normalised longitudinal compressive stress.

Based on the parametric studies conducted for circumferential interacting defects subjected to combined loadings, it was observed that at a constant effective defect length and longitudinal compressive stress, the increase in normalised defect spacing from 0.00 to 2.00 caused an insignificant increase in the normalised failure pressure of the pipe for normalised effective defect depths of 0.05 to 0.80, as shown in Figure 9.

**Figure 10:**
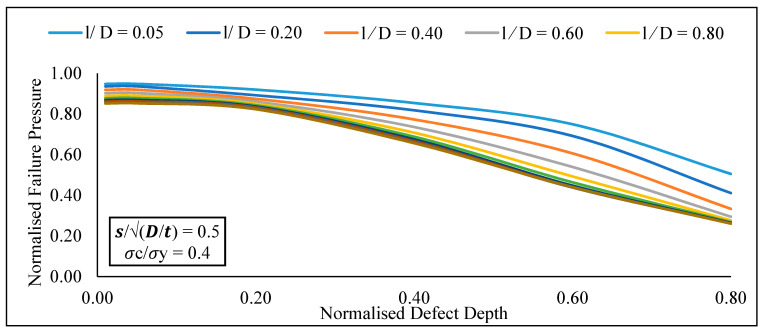
Normalized failure pressure against normalized defect depths for various normalized defect lengths at constant normalized defect spacing and normalized longitudinal compressive stress.

**Figure 11:**
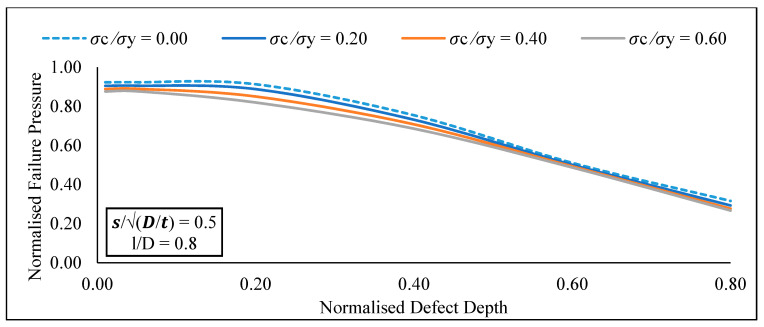
Normalized failure pressure against normalized defect depths for various longitudinal compressive stresses at a constant normalized defect spacing and normalized defect length.

At a constant longitudinal compressive stress and defect spacing, the increase in normalised effective defect length from 0.05 to 1.20 significantly reduces the normalised failure pressure of a pipe for normalized effective defect depths of 0.20 to 0.80 (Figure 10). Furthermore, at a constant defect spacing and effective defect length, the increase in normalised effective defect depth from 0.05 to 0.80 significantly influences the normalised failure pressure of the pipe for normalised longitudinal compressive stress values of 0.00 to 0.80 (Figure 11). At a constant defect length and spacing, the increase in normalised longitudinal compressive stress from 0.00 to 0.80 insignificantly influences the normalised failure pressure of a pipe for normalized defect depths of 0.05 to 0.80.

## 4. Recommendations for Future Studies

The current equation is limited only to the prescribed range and material. Future studies should consider different types of material. To create a robust artificial neural network, more training data covering greater parameter variations should be considered during the generation of the ANN data.

## 5. Conclusions

Current corrosion assessment methods are not applicable to circumferentially aligned interacting corrosion defects subjected to internal pressure and longitudinal compressive stress. This study proposes an empirical equation to predict the failure pressure of a pipe with circumferentially interacting corrosion defects subjected to internal pressure and longitudinal compressive stress as a function of normalised defect spacing, defect depth, defect length, and longitudinal compressive stress. The new equations predicted failure pressures for API 5L X80 pipes with an R^2^ value of 0.9999 and an error range of −9.99% to 0.80% for normalised defect spacings ranging from 0.00 to 2.00, normalised defect depths ranging from 0.00 to 0.80, normalised defect lengths ranging from 0.00 to 1.20, and normalised longitudinal compressive stress ranging from 0.00 to 0.80.

Following that, an extensive parametric study was carried out utilising the proposed model to demonstrate a correlation between defect geometries and failure pressure of API 5L X80 pipes. Based on the findings conducted with the newly established assessment approach for circumferential interacting defects subjected to combined loading, it is possible to conclude:

- At a constant effective defect length and longitudinal compressive stress, there was insignificant increase in the normalised failure pressure of the pipe for normalised effective defect depths of 0.05 to 0.80 with an increase in normalised defect spacing from 0.00 to 2.00.
- At a constant longitudinal compressive stress and defect spacing, the normalised failure pressure of a pipe significantly reduces, for normalized effective defect depths of 0.20 to 0.80 with an increase in normalised effective defect length from 0.05 to 1.20
- At a constant defect spacing and effective defect length, the normalised failure pressure of the pipe significantly reduces for normalised longitudinal compressive stress values of 0.00 to 0.80 with an increase in normalised effective defect depth from 0.05 to 0.80.
- At a constant defect length and spacing, there was insignificant change in the normalised failure pressure of a pipe for normalized defect depths of 0.05 to 0.80 when the normalised longitudinal compressive stress increased from 0.00 to 0.80 insignificantly.

## Figures and Tables

**Table 1:** Interaction rules applied in common corrosion assessment methods [31].

Corrosion Assessment Code	Interaction Rule
DNV-RP-F101	${\left(s_{c}\right)}_{Lim}=\pi \sqrt{Dt}$
API 579	${\left(s_{c}\right)}_{Lim}=\left(w_{1}+w_{2}\right)/2$
BS 7910	${\left(s_{l}\right)}_{Lim}=3.0\sqrt{Dt}$

**Table 2:** Geometric parameters for finite element analysis (FEA) parametric study.

Input Parameters	Value (s)
Pipe material	API 5L X80
Outer diameter of pipe, D (mm)	300
Wall thickness, t (mm)	10
Straight pipe length, L (mm)	2000
$\mathrm{Normalized}\;\mathrm{defect}\;\mathrm{spacing},\;s_{c}/\sqrt{Dt}$	0.0, 0.5, 1.0, 2.0
Normalized defect length, l/D	0.0, 0.2, 0.4, 0.6, 0.8
Normalized defect depth, d/t	0.0, 0.2, 0.4, 0.6, 0.8
Normalized longitudinal compressive stress, *σ_c_/σ_y_*	0.0, 0.2, 0.4, 0.6, 0.8

**Table 3:** Model features and justification.

Model Feature	Justification
Quarter model	Reduction in FEA computation time without compromising on the accuracy of the predictions [32].
Pipe full length set to 2000 mm	Prevention of end cap effects [26].
Endplates of 20 mm	Enables longitudinal compressive stress to be distributed evenly across the pipe wall [43].
Rectangular-shaped corrosion defect idealization	Results in safer lower bound failure pressure without compromising the accuracy of the predictions [1,29,44,45].

**Table 4:** Convergence test results for the optimal number of mesh layers at the defect region for Test Model 1.

Number of Element Layers	$\mathrm{Normalized}\;\mathrm{Failure}\;\mathrm{Pressure},\;P_{f}/P_{i}$
1	0.92
2	0.93
**3**	**0.95**
4	0.95
5	0.95

**Table 5:** Corrosion defect parameters for full scale burst tests by Bjorney et al. [50].

Grade	Specimen	*d* (mm)	*l* (mm)	*w* (mm)	*σ_l_* (MPa)
X52	Test 1	5.15	243	154.5	0.0
	Test 5	3.09	162	30.9	48.0
	Test 6	3.09	162	30.9	84.0

**Table 6:** Finite element method (FEM) validation against full scale burst tests by Bjorney et al. [50].

Specimen	Burst Pressure (MPa)	FEA Failure Pressure (MPa)	Percentage Difference (%)
Test 1	23.2	22.95	−1.08
Test 5	28.6	28.35	−0.87
Test 6	28.7	27.00	−5.92

**Table 7:** Corrosion defect parameters for full scale burst tests by Benjamin et al. [51].

Grade	Specimen	d (mm)	l (mm)	w (mm)	$s_{l}\;(\mathrm{mm})$	$s_{c}\;(\mathrm{mm})$
X80	IDTS 2	5.39	39.6	31.9	0.0	0.0
	IDTS 3	5.32	39.6	31.9	20.5	0.0
	IDTS 4	5.62	39.6	32.0	0.0	9.9

**Table 8:** FEM validation against full scale burst tests by Benjamin et al. [51].

Burst Pressure (MPa)	FEA Failure Pressure (MPa)	Percentage Difference (%)
22.68	22.40	−1.23
20.31	20.12	−0.94
21.14	20.62	−2.46

**Table 9:** Mechanical properties of API 5L X80.

Material Properties	Value
Modulus of elasticity, *E* (GPa)	210.0
Poisson’s Ratio, υ	0.3
Yield strength, *σ_y_* (MPa)	531.0
UTS, *σ_u_* (MPa)	655.0
True UTS, σ*_u_ (MPa)	718.2

**Table 10:** Training data generated for the development of artificial neural network (ANN).

$s_{c}/\sqrt{Dt}$	${\left(d/t\right)}_{e}$	${\left(l/D\right)}_{e}$	$\sigma_{c}/\sigma_{y}$			
			0.00	0.20	0.40	0.60
0.00	0.00	0.00	1.00			
0.00	0.20	0.20	0.92	0.91	0.89	0.84
		0.40	0.91	0.90	0.88	0.82
		0.60	0.88	0.87	0.86	0.79
		0.80	0.87	0.86	0.85	0.79
	0.40	0.20	0.84	0.83	0.80	0.74
		0.40	0.74	0.73	0.72	0.71
		0.60	0.72	0.71	0.69	0.67
		0.80	0.70	0.69	0.66	0.65
	0.60	0.20	0.72	0.71	0.70	0.66
		0.40	0.61	0.59	0.57	0.54
		0.60	0.51	0.50	0.50	0.49
		0.80	0.51	0.50	0.49	0.47
	0.80	0.20	0.55	0.53	0.51	
		0.40	0.37	0.36	0.36	
		0.60	0.31	0.30	0.28	
		0.80	0.30	0.29	0.26	
0.50	0.20	0.20	0.94	0.92	0.89	0.85
		0.40	0.92	0.91	0.88	0.83
		0.60	0.88	0.87	0.86	0.80
		0.80	0.87	0.86	0.85	0.78
	0.40	0.20	0.85	0.84	0.80	0.75
		0.40	0.74	0.73	0.72	0.70
		0.60	0.72	0.71	0.69	0.67
		0.80	0.71	0.70	0.66	0.64
	0.60	0.20	0.72	0.71	0.70	0.65
		0.40	0.59	0.58	0.57	0.55
		0.60	0.52	0.51	0.50	0.48
		0.80	0.52	0.51	0.49	0.46
	0.80	0.20	0.54	0.53	0.51	
		0.40	0.38	0.36	0.36	
		0.60	0.31	0.30	0.28	
		0.80	0.29	0.28	0.26	
1.00	0.20	0.20	0.93	0.92	0.89	0.84
		0.40	0.91	0.90	0.88	0.82
		0.60	0.87	0.86	0.86	0.80
		0.80	0.86	0.85	0.85	0.78
	0.40	0.20	0.85	0.84	0.80	0.75
		0.40	0.73	0.72	0.72	0.71
		0.60	0.73	0.71	0.69	0.66
		0.80	0.70	0.69	0.66	0.64
	0.60	0.20	0.72	0.71	0.70	0.66
		0.40	0.59	0.58	0.57	0.55
		0.60	0.51	0.50	0.50	0.49
		0.80	0.51	0.50	0.49	0.47
	0.80	0.20	0.54	0.53	0.51	
		0.40	0.37	0.36	0.36	
		0.60	0.31	0.30	0.28	
		0.80	0.29	0.28	0.26	
2.00	0.20	0.20	0.93	0.92	0.89	0.84
		0.40	0.91	0.90	0.88	0.83
		0.60	0.88	0.87	0.86	0.80
		0.80	0.86	0.85	0.85	0.79
	0.40	0.20	0.85	0.84	0.80	0.75
		0.40	0.74	0.73	0.72	0.70
		0.60	0.71	0.70	0.69	0.66
		0.80	0.70	0.69	0.66	0.64
	0.60	0.20	0.73	0.71	0.70	0.65
		0.40	0.59	0.58	0.57	0.55
		0.60	0.52	0.51	0.50	0.48
		0.80	0.51	0.50	0.49	0.47
	0.80	0.20	0.55	0.54	0.51	
		0.40	0.37	0.35	0.36	
		0.60	0.30	0.29	0.28	
		0.80	0.29	0.28	0.26	

**Table 11:** Coefficient of determinant of the developed ANN models.

Model	No. of Hidden Layers	No. of Neurons in Hidden Layer 1	No. of Neurons in Hidden Layer 2	No. of Neurons in Hidden Layer 3	R^2^ Value
CID1	1	1	-	-	0.92
CID2	1	2	-	-	0.93
CID3	1	3	-	-	0.93
CID4	1	4	-	-	0.94
CID5	2	4	1	-	0.96
CID6	2	4	2	-	0.98
**CID7**	**2**	**4**	**3**	**-**	**0.99**
CID8	2	4	4	-	0.99
CID9	3	4	4	1	0.99
CID10	3	4	4	2	0.98
CID11	3	4	4	3	0.95
CID12	3	4	4	4	0.90

**Table 12:** Comparison of the intact pressure values of the pristine pipe.

Maximum Hoop Stress Theory [A] (MPa)	FEM [B] (MPa)	Newly Developed Method [C] (MPa)	Percentage Difference between [A] and [C] (%)	Percentage Difference between [B] and [C] (%)
51.30	50.94	51.29	−0.02	0.68

**Table 13:** Comparison of the failure pressure obtained using FEM and the empirical equation.

$s/\sqrt{D/t}$	${\left(l/D\right)}_{e}$	${\left(d/t\right)}_{e}$	σc/σy	$P_{nf,FEM}$	$P_{nf,Eq}$	Percentage Difference (%)
0.25	0.65	0.09	0.30	0.95	0.94	−1.16
0.25	0.65	0.09	0.60	0.86	0.84	−1.38
0.25	1.45	0.10	0.30	0.93	0.87	−6.35
0.25	1.85	0.10	0.30	0.92	0.88	−4.10
0.25	1.85	0.10	0.60	0.85	0.80	−6.17
0.25	0.65	0.28	0.30	0.84	0.80	−4.28
0.25	0.65	0.28	0.60	0.78	0.76	−1.99
0.25	1.45	0.34	0.60	0.70	0.68	−2.59
0.25	1.45	0.44	0.60	0.60	0.60	−0.41
0.25	1.45	0.48	0.30	0.59	0.58	−1.69
0.25	0.65	0.51	0.60	0.63	0.62	−1.19
0.25	1.45	0.53	0.60	0.52	0.51	−2.28
0.25	1.45	0.77	0.50	0.27	0.25	−7.57
0.25	1.45	0.77	0.60	0.25	0.24	−2.55
0.30	1.05	0.47	0.30	0.62	0.60	−2.93
0.30	0.65	0.27	0.30	0.85	0.81	−4.59
0.40	1.47	0.43	0.60	0.60	0.60	0.19
0.40	1.87	0.53	0.50	0.52	0.50	−3.21
0.70	0.73	0.37	0.30	0.74	0.71	−3.40
0.70	1.53	0.50	0.60	0.52	0.52	−0.84
0.70	1.93	0.75	0.50	0.26	0.24	−7.60
0.90	0.76	0.43	0.50	0.65	0.63	−2.88
0.90	1.56	0.20	0.30	0.87	0.82	−5.99
1.00	0.58	0.40	0.20	0.73	0.71	−2.83
0.50	0.89	0.60	0.40	0.49	0.48	−2.52
0.80	1.75	0.30	0.70	0.68	0.65	−4.10
0.20	0.45	0.20	0.30	0.91	0.88	−3.62
0.30	0.65	0.40	0.60	0.70	0.68	−2.37
0.40	1.45	0.60	0.30	0.47	0.44	−5.70
0.80	1.05	0.30	0.60	0.73	0.72	−1.57
0.90	0.65	0.20	0.30	0.90	0.85	−5.42
1.00	1.47	0.40	0.60	0.62	0.61	−2.30
1.20	1.87	0.60	0.40	0.39	0.39	−0.89
0.50	0.60	0.40	0.30	0.75	0.73	−2.32
1.40	1.53	0.30	0.20	0.74	0.72	−2.64

## Data Availability

Not applicable.

## Nomenclature

ANSYS	ANSYS 16.1 Structural Product of Mechanical ANSYS Parametric Design Language (APDL)
ANN	Artificial neural network
DNV	DNV-RP-F101 corrosion assessment method
DOF	Degrees of freedom
FEA	Finite element analysis
FEM	Finite element method
UTS	Ultimate tensile strength
D	Diameter of pipe
*E*	Modulus of elasticity
L	Length of pipe
$N_{h}$	Number of neurons in hidden layer
$N_{i}$	Number of input neuron
$N_{o}$	Number of output neuron
$P_{i}$	Intact pressure of pipe
$P_{f}$	Failure pressure of corroded pipeline
$P_{nf,Eq}$	Normalised failure pressure using developed equation
$P_{nf,FEM}$	Normalised failure pressure using FEM
$h_{x,y}$	Neuron in hidden layer
$i_{n}$	Normalised input variable
d	Defect depth
l	Defect length
$r_{i}$	Internal radius of pipe
$s_{c}$	Circumferential spacing between corrosion defects
$s_{l}$	Longitudinal spacing between corrosion defects
t	Thickness of pipe
o	Output variable
$o_{n}$	Normalised output variable
w	Defect width
${\left(d/t\right)}_{e}$	Effective normalised defect depth
${\left(l/D\right)}_{e}$	Effective normalised defect length
${\left(s_{c}\right)}_{Lim}$	Circumferential interaction limit
${\left(s_{l}\right)}_{Lim}$	Longitudinal interaction limit
*σ_e_*	Effective von Mises stress
*σ_h_*	Hoop stress
*σ_l_*	Longitudinal stress
*σ_r_*	Radial stress
*σ_y_*	Yield stress
*σ_UTS_*	Ultimate tensile strength
*σ*_UTS_*	True ultimate tensile strength
*υ*	Poisson’s Ratio
